# The Impact of Early Neuroimaging and Developmental Assessment in a Preterm Infant Diagnosed with Cerebral Palsy

**DOI:** 10.1155/2019/9612507

**Published:** 2019-02-07

**Authors:** Lily Gullion, Jennifer Stansell, Hunter Moss, Dorothea Jenkins, Turki Aljuhani, Patty Coker-Bolt

**Affiliations:** ^1^Division of Occupational Therapy, Medical University of South Carolina, Charleston, SC, USA; ^2^Graduate Studies, Medical University of South Carolina, Charleston, SC, USA; ^3^Division of Pediatrics-Neonatology, Medical University of South Carolina, Charleston, SC, USA

## Abstract

Premature infants are at risk for cerebral palsy (CP) that is typically diagnosed between 18–24 months. We present a case study of an infant who was discharged from the neonatal intensive care unit (NICU) without obvious neurological deficits but was later diagnosed with hemiplegic CP. The infant was enrolled in an infant motor study, which included neuroimaging and developmental motor assessments. At term, anatomical MRI showed bilateral periventricular leukomalacia, abnormal brain metabolites in frontal white matter via MR spectroscopy (MRS), and low fractional anisotropy (FA) values obtained from diffusional kurtosis imaging (DKI) in several cortical white matter tracts compared to a group of typically developing infants without neuroimaging abnormalities. In addition, the infant scored below average on a developmental assessment administered at term and three months as well as on the standard Bayley III assessment at 12 months. Abnormal neuroimaging and low scores on the early developmental assessment prompted referral for intervention services at two months. With intensive therapy, by 45 months, the infant was average in self-care, mobility, and communication skills, although below average in visual motor and gross motor coordination. This case highlights the clinical impact of early detection and referral using combined neuroimaging and developmental testing.

## 1. Introduction

In the US, one in every 10 infants is born prematurely at less than 37 weeks gestational age (GA) [1]. Preterm infants are at risk for brain injury and cerebral palsy (CP) [2], which is reliably diagnosed at 18–24 months, when atypical movements and delayed motor skills become evident [3, 4]. Other than CP, preterm infants are additionally at risk for delays in motor, language, or cognitive developmental outcomes with less than half of them having been identified by kindergarten [4, 5]. Therefore, there is a significant gap in the early identification of delays that could initiate earlier intervention services such as occupational and physical therapies; however, these are not routinely initiated until later in the first or second years of life [6–8]. According to the most recent recommendations for evidence-based identification and treatment of CP, intervention should be implemented as soon as possible to optimize recovery from brain injury or dysmaturity associated with preterm birth [8, 9]. Although rigorous research on the outcomes of early intervention programs is limited to date, early therapy programs may take advantage of infant neuroplasticity, facilitate learning motor movements, and potentially improve motor, language, and cognitive outcomes [9].

Neuroimaging is qualitatively sensitive in identifying brain lesions in infants eventually diagnosed as having CP well before clinical deficits are apparent. Neuroimaging modalities such as magnetic resonance spectroscopy (MRS) and diffusional kurtosis imaging (DKI) [10] are able to quantify metabolite concentrations and the non-Gaussian self-diffusion of water, respectively. By quantifying the concentration of free-tumbling moieties in a specific brain region, MRS can assess oxidative stress and identify biochemical markers of injury [11, 12]. DKI measures the water diffusion displacement probability density function in tissue, and the resulting parametric diffusion metrics relate to the integrity of tissue microstructures. One such metric is the fractional anisotropy (FA) which contains information about the anisotropy of the water diffusion along axons, with values closer to the one indicating greater axonal integrity [13]. Combining developmental assessments with neuroimaging biomarkers such as DKI-derived FA and MRS metabolite ratios could provide earlier and more accurate detection of infants who will develop motor delays leading to earlier referrals for therapy services [8, 14, 15].

The purpose of this report is to examine a case of significant but occult white matter injury in a preterm infant. This report is also unique in documenting both the early term age neuroimaging and motor tests, and the effect of interventions at an extended follow-up at 45 months of age. We found that DKI-derived FA values and MRS metabolite ratios in areas distant from the areas of white matter injury in the brain were different in the case from typically developing infants; further, they were associated with scores on developmental assessments at term, three-month, and one-year corrected age (CA). This case highlights specific neuroimaging abnormalities at term CA by elucidating what these findings may mean in the context of future development and the impact of early intervention.

## 2. Case Description

This study involved a preterm infant born at 31 weeks GA with APGAR scores of five at one minute and eight at five minutes. The pregnancy was complicated by twin-twin transfusion syndrome and maternal chorioamnionitis. The infant's birthweight was 1325 grams. Initial respiratory effort at birth was poor, and the infant required positive pressure ventilation and surfactant for respiratory distress syndrome. The infant was weaned to room air on the third day of life and received seven days of antibiotics for sepsis. The infant was discharged taking all feeds by mouth at 36 weeks GA, weight was 2251 grams, and there were no apparent neurological issues. We obtained consent from the infant's family to enroll the infant in an early motor study of preterm infants and approved by the institutional review board at the Medical University of South Carolina (MUSC). As part of participation in this research study, we performed neuroimaging with motor assessments at term GA and developmental assessments at three-month and one-year CA. In addition, we performed a follow-up developmental assessment and quantitative interview with the infant's family at 45 months prior to the child's enrollment in a therapeutic constraint-induced movement therapy program at the MUSC not related to the original infant motor study.

## 3. Neuroimaging and Developmental Assessments

Unsedated MRI scans that included MRS and DKI were obtained at a 41 weeks GA using a 3T MRI system (Siemens Tim Trio), as previously described [16]. For MRS, 1H-MR spectra were acquired using point-resolved spectroscopy (PRESS), relaxation time of 1500 ms, and echo times (TE) of 30 ms and 270 ms, with 128 signal averages. A single voxel (15 × 15 × 15 mm) was placed in the left basal ganglia (BG) and in the right frontal white matter (WM). MRS spectra were fit using a LCModel [17] with a simulated basis set generated using VeSPA [18]. Standard deviation (SD) < 20% was used for inclusion of metabolite concentrations [19]. The concentration of major metabolites N-acetylaspartate (NAA), phosphorylcholine plus glycerophosphocholine (Cho), glutamate plus glutamine (Glx), and creatine plus phosphocreatine (Cr) were measured at both TE of 30 and 270 ms, while myo-inositol (mI) was only measured at 30 ms. Ratios of metabolite concentrations obtained at the same echo times were used for analysis, and all metabolites were quantified as ratios relative to Cr (e.g., NAA/Cr divided by Cho/Cr to obtain NAA/Cho).

For DKI, data was gathered using a twice-refocused DWI sequence to suppress eddy current distortion [20]. Three b-values were gathered at *b* = 0, 1000, and 2000 s/mm^2^ with 64 diffusion encoding directions for the two nonzero b-values. Other imaging parameters were echo time (TE) = 99 ms, repetition time (TR) = 4800 ms, with full Fourier space encoding, and a voxel size = 2.7 mm isotropic. With the same imaging parameters, a separate nondiffusion weighted (*b* = 0 s/mm^2^, b0) scan was performed immediately following the DKI scan that acquired 9 additional b0 images to improve the signal estimation. The raw images were then denoised [21], corrected for the Rician noise bias [22], and the Gibbs ringing artifact correction was applied [23]. The image voxels were each then smoothed using a Gaussian kernel of 1.25 times the voxel size. To obtain diffusion metrics, a diffusional kurtosis estimator (DKE) was used to derive parametric diffusion maps [24]. Data from this infant's neuroimaging and developmental assessments were plotted and compared against their MRS ratios, white matter FA, and developmental outcomes with other infants enrolled in the same early motor development study.

The Specific Test of Early Infant Motor Performance (STEP) was used for early motor assessment at term and three-month CA. The STEP is a novel developmental screening test for preterm infants designed for rapid and early detection of abnormalities of tone and movement patterns [25, 26]. The STEP test consists of ten of the most sensitive motor movements from the Test of Infant Motor Performance, which we rescaled for better sensitivity and then validated with motion kinematics, correlating the angles we use in scoring with neuroimaging and longer-term outcomes [16, 19, 26–28]. We also performed the Bayley III, as the gold standard neurodevelopmental assessment for infants at risk for motor, language, or cognitive delays, at 12-month CA [29, 30]. For later assessments at 45 months, we performed the Peabody Developmental Motor Scales-2 (PDMS-2) and the Pediatric Evaluation of Disability Inventory (PEDI). The PDMS-2 is a standardized, norm-referenced assessment, which evaluates gross and fine motor skills with subtests such as locomotion, object manipulation, grasping, and visual motor integration in children aged birth to six years of age [31]. The PEDI is a standardized assessment for children aged 6 months to 7.5 years, which measures functional performance in the areas of self-care, mobility, and social function based on the clinical judgment or parent report questionnaire. The PDMS-2 and the PEDI evaluate different functional aspects but are strongly correlated, indicating that the use of both assessments may allow for a complementary and more complete profile of the child than can be provided using one instrument alone [32]. The assessments were administered by one trained examiner.

### 3.1. MRI Results and Early Referral

**Figure 1:**
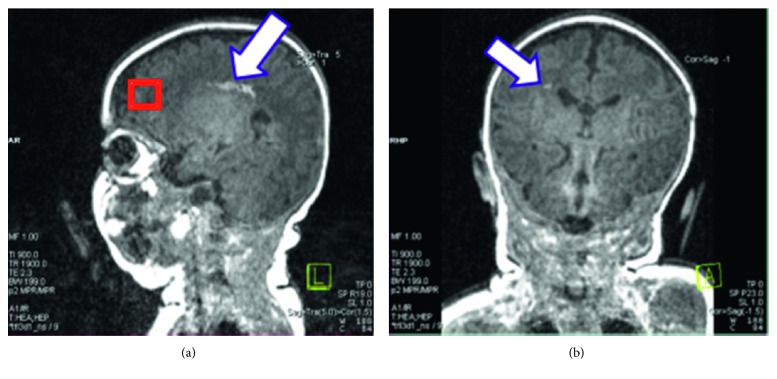
Infant's MRI results reveal periventricular leukomalacia in both the left (a) and right (b) sides of brain.

The infant's MRI images revealed a bilateral periventricular leukomalacia (PVL) (Figure 1), with greater involvement of right than left parietal white matter. Study investigators immediately referred the infant, who received early intervention services at two months of age, in addition to the usual high-risk clinic visit and close general pediatric follow-up. The infant was diagnosed with hemiplegic CP at nine months of age.

### 3.2. Developmental Assessment Results

The case infant's scores were below average at term, three-month, and 12-month CA on the STEP and Bayley III motor and cognitive domains, respectively (Table 1). The infant received early intervention and occupational and physical therapy services based on the results of these early assessments and neuroimaging findings. These early therapies, started as early as two months of age, included goal-directed therapy and constraint-induced movement therapy later in the first year of life for two to four hours per week, which targeted arm movements to improve skill in age-appropriate developmental tasks (ie., self-care, early mobility, play, and exploration of environment). At the follow-up assessment at 45 months of age using the PDMS-2 and the PEDI developmental assessments, the case infant's scores were average in self-care, functional mobility, and social communication skills. She could complete basic self-care tasks for her age including self-feeding using a fork and spoon and self-dressing including putting on shirts, pants, socks, and shoes. She was also able to ambulate independently, follow simple directions, and communicate with family and peers. The improvements seen in self-care, early mobility, and social and communication skills indicate that early, evidence-based therapies had a positive impact on her overall development. Scores on the PDMS-2 related to visual motor integration and gross motor coordination, such as cutting with scissors, copying a circle, running on uneven surfaces, or catching a ball, remained below average (Table 1). In addition, her parent noted continued concerns with sustained attention to tasks.

### 3.3. MRS Results

**Figure 2:**
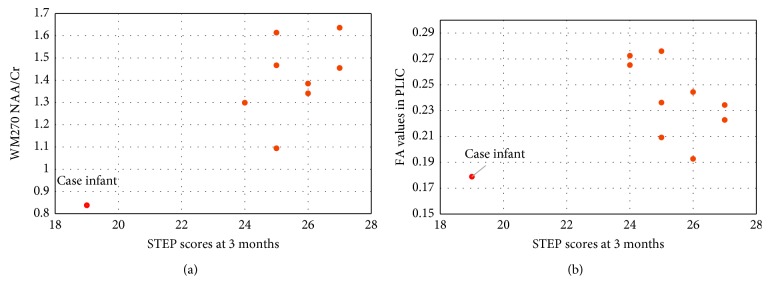
(a) Case infant's WM NAA/Cr ratio at TE = 270 ms and STEP scores versus infants with typical motor skill development. (b) Case infant's FA values in PLIC and STEP scores versus infants with typical motor skill development.

An analysis of MRS data revealed abnormal brain metabolites in both the frontal WM and the BG. The red box in Figure 1 shows the MR voxel placement in the frontal white matter where brain metabolites were measured in this infant. Because voxel placement is distant from the obvious site of injury in the parietal lobe, the results suggest a global rather than focal insult and neuronal injury. In healthy neuronal cell bodies and axons, the ratios of N-acetylasparte to creatine, NAA/Cr, should increase with development [33, 34]. This infant displayed a decreased NAA/Cr (Figure 2(a)), indicating a decreased frontal white matter neuronal and axonal integrity compared to other infants enrolled in the same early motor developmental study who had average motor developmental scores.

### 3.4. DKI Results

**Figure 3:**
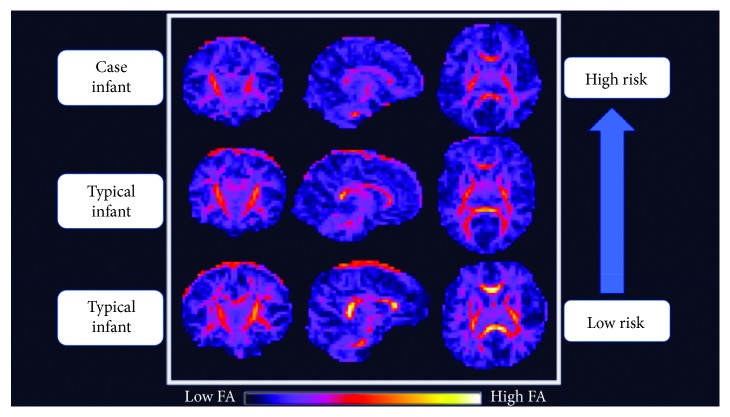
Qualitative FA comparison of the case infant (high risk for motor delay) and peers (low risk for motor delays) based on STEP assessment scores.

In Figure 2(b), FA values in the posterior limb of the internal capsule (PLIC) are compared to STEP scores at 3 months. The case infant showed qualitatively low FA values compared to typically developing infants without WM injury, in the corticospinal tract (CST), inferior fronto-occipital fasciculus (IFOF), posterior limb of the internal capsule (PLIC), and posterior thalamic radiations (PTR). These tracts are associated with visual perception skills, attention, cognition, and motor coordination [13, 14, 35, 36]. A qualitative comparison was completed using the case infant's FA values at the time of scan, and two infants in the study with typical motor scores on the STEP assessment who were at low risk for later motor delays (Figure 3). Both low-risk infants with typical motor scores on the STEP had higher FA values in the genu and the splenium of their corpus callosum, while the case infant has low FA values in these areas. The higher FA values are an indication of more robust axonal organization and integrity [15].

## 4. Discussion

This case study examined the implications of the early detection of CP through a combination of early neuroimaging using MRS and DKI combined with motor assessment. This case infant was discharged from the newborn nursery with a normal neurological exam but was later identified with occult white matter injury and subsequent developmental delays after enrolling in an infant motor research study. Participation in this research study and the use of early neuroimaging in combination with the motor assessment helped to identify this infant as at-risk for delays which led to early referral to therapy services. In addition, this case study is one of a few studies that relate early MRS- and DKI-derived results with short- and long-term developmental screening. Future studies will be done to assess the utility of the kurtosis metrics that set DKI apart from the more conventional diffusion tensor imaging (DTI). Not only is DKI more accurate than DTI, the kurtosis metrics also are of interest since they have been shown in other neuropathologies to be more closely related to microstructural integrity [37]. In addition, the infant's DKI scans at term revealed the difference between the case infant and low-risk infants before any clinical motor deficits were apparent.

In this case, the MRS metabolites and DKI-derived FA values in early neuroimaging at term are associated with the deficits seen clinically later in life including visual motor skills, attention to task, and motor incoordination. Additionally, the abnormal brain metabolites in frontal WM, distant to the site of PVL, indicate this infant, such as many preterm infants, may have global brain injury although head ultrasound or gross MRI findings may be focal [12, 16]. The MRS findings in this case included decreased NAA/Cr ratios which are consistent with research on long-term development after significant brain injury in preterm infants [38]. NAA decreases with both acute and chronic neuronal cell death, and decreased NAA/Cr ratios in the BG and WM have been shown to be predictive of later developmental delay and poor developmental outcomes in preterm infants [33, 39]. Lower NAA in the BG may reflect neural metabolic impairment, lower neuronal density, or cell body volume and may be associated with decreased neuronal axonal integrity in neonatal white matter injury [33, 38].

In this case, early therapies had the most impact on self-care, mobility and ambulation, and social and communication skills, the domains which are critically important in the first three years of life. Future studies could include a clinical trial to determine the benefits of early therapies such as those used for the case infant that targeted visual motor skills required for school-age tasks and motor coordination required for safe participation in higher level motor skills such as jumping and multistep gross motor play on outdoor playground equipment. Larger trials could also use historical controls with neuroimaging (MRI/MRI/DTI or DKI) paired with motor assessments, such as the STEP, to determine the benefits of early therapies for infants with neurological conditions. In conclusion, this case report reinforces the recent recommendations on the use of neuroimaging in combination with early motor assessments to identify infants at risk for CP before six-month CA [9]. Early diagnosis could become the standard of care, with early referral to therapies that may optimize neuroplasticity and functional outcomes within the first year of life.

## Figures and Tables

**Table 1:** Results of developmental assessments.

Assessment	Age (corrected age, CA)	Subtest	Score	Interpretation
STEP	Term		14	At risk
	3 months		19	At risk

TIMP	Term		40	At risk
	3 months		89	At risk

Bayley III	12 months	Cognition scaled score	10	At risk
		Receptive language score	7	At risk
		Expressive language score	7	At risk
		Fine motor scaled score	8	At risk
		Gross motor scaled score	4	At risk
		Language composite score	83	Normal
		Language summary score	14	Normal
		Motor summary score	12	Normal
		Motor composite score	76	Normal
		Cognition composite score	100	Normal

PDMS-2	45 months	Reflexes		N/A
		Stationary movement	41	Below average
		Locomotion	129	Below average
		Object manipulation	26	Below average
		Grasping	39	Very poor
		Visual motor integration	98	Poor

PEDI—Functional scales	45 months	Self-care	55	Average
		Mobility	59	Average
		Social function	51	Average

STEP, Specific Test of Early Infant Motor Performance; TIMP, Test of Infant Motor Performance; PDMS-2, Peabody Developmental Motor Scales-2; PEDI, Pediatric Evaluation of Disability Inventory.
